# Concentration of novel urinary tract infection biomarkers in neonates

**DOI:** 10.1038/s41598-024-53486-2

**Published:** 2024-02-06

**Authors:** Maria Jebbia, Sudipti Gupta, Brett G. Klamer, Leeann Pavlek, Christina B. Ching, Tahagod H. Mohamed, Brian Becknell

**Affiliations:** 1 The Kidney and Urinary Tract Center at Nationwide Children’s Hospital, 700 Children’s Dr, Columbus, OH USA; 2 Division of Perinatal Medicine, Nationwide Children’s Hospital, Columbus, OH USA; 3 Center for Clinical and Translational Research, The Abigail Wexner Research Institute at Nationwide Children’s Hospital, Columbus, OH USA; 4 Biostatistics Resource at Nationwide Children’s Hospital, Columbus, OH USA; 5 Center for Biostatistics, The Ohio State University, Columbus, OH USA; 6 Division of Pediatric Urology, Department of Surgery, Nationwide Children’s Hospital, Columbus, OH USA; 7 Division of Nephrology and Hypertension, Nationwide Children’s Hospital, Columbus, OH USA

**Keywords:** Urinary tract infections, UTI, Neonates, Biomarkers, Antimicrobial peptides, AMPs, Kidney, Kidney diseases, Nephrology

## Abstract

Urinary tract infections (UTIs) are a common comorbidity in hospitalized neonates. The current UTI diagnostics have several limitations including invasive collection of urinary samples to ensure sterility, risk of contamination and lack of consensus definitions of UTI based on urine culture. Antimicrobial peptides (AMPs) have been recently utilized as novel biomarkers that can efficiently and accurately diagnose pediatric UTI. However, the concentration of AMPs in neonatal urine is not well-defined. Urine from neonates admitted to a single level IV neonatal intensive care unit was obtained to determine baseline concentration of two AMPs, Ribonuclease 7 (RNase 7) and Beta Defensin-1 (BD-1) and to define the relationship between AMP concentration and gestational age (GA). AMP levels were normalized to urine creatinine. RNase 7 and BD-1 were expressed in neonatal urine (n = 66) regardless of GA and as early as 22 weeks gestation. Urinary concentrations of both AMPs decreased as GA and birthweight increased. The overall median urinary RNase 7/UCr and BD-1/UCr values were 271 ng/mg, and 116 ng/mg, respectively. Median urinary concentrations of RNase 7/UCr for infants born at < 27, 27–32, 33–35 and ≥ 36 weeks were 569, 308, 254, and 124 ng/mg respectively. Similarly, the concentrations of BD-1/UCr at these GA were 166, 115, 108, and 14 ng/mg, respectively. Baseline neonatal urinary concentration of two AMPs (RNase 7 and BD-1) and the variation by GA were identified. This is an essential first step toward the potential utilization of AMPs in improving neonatal UTI diagnostics.

## Introduction

Urinary tract infections (UTIs) including pyelonephritis are a common comorbidity in neonates admitted to the neonatal intensive care unit (NICU). The prevalence of UTIs among febrile full-term neonates ranges from 7 to 15%^1,2^ and increases with decreasing GA^2^. UTIs could lead to significant comorbidities including future chronic kidney disease and hypertension^3,4^. Currently the gold standard for UTI diagnosis is urine culture obtained sterilely via bladder or suprapubic puncture^5^; however, this approach has several limitations. First, to obtain sterile samples, urine is collected via invasive procedures such as urethral catheterization or suprapubic bladder aspiration which do not completely eliminate the risk of contamination when appropriate skin cleansing is not performed. Second, urethral catheterization can be very difficult in extremely preterm and very low birth weight infants. Third, there is no standardized UTI definition based on urine cultures leading to laboratory variations in the cutoff bacterial colony counts that meet UTI criteria. Therefore, the current UTI diagnostics pose several challenges especially in the neonatal period.

Antimicrobial peptides (AMPs) are small, cationic peptides that constitute a fundamental component of the innate immune system. AMPs are synthesized by white blood cells and epithelial cells constitutively or upon stimulation by pathogens^1,6–8^. The most studied AMPs of the human genitourinary (GU) tract are Ribonuclease (RNase) 7, neutrophil gelatinase-associated lipocalin (NGAL) and Beta Defensin 1 (BD-1)^9–13^. RNase 7 is a member of the RNase A superfamily and was first purified from human skin in 2002 by Harder and Schroder^11^. Spencer and colleagues demonstrated the presence and antibacterial properties of RNase 7 in the urine and genitourinary tract in pediatric patients^13^. BD-1, a member of the beta defensin class of AMPs, was first isolated from human plasma, but the GU tract and the kidney in particular is its primary source^8,14–16^. Our group characterized the presence and antibacterial activity of BD-1 in the human and murine GU tract^14^.

AMPs have been recently utilized as novel biomarkers that can efficiently and accurately diagnose pediatric UTI^17^ and showed a potential to overcome the limitations of current UTI diagnostics. AMPs have been characterized in the GU tract of pediatric and adult patients^1,13,14^; however, it is unknown whether AMPs are present in the neonatal GU tract. Moreover, the effects of GA and birthweight on the concentration of AMPs are not characterized. Therefore, we designed this study to (i) evaluate the concentration of AMPs in neonatal urine and (ii) define the effects of GA and birthweight on AMP concentration.

## Methods

### Patient population

The study was performed at a level IV NICU at the Nationwide Children’s Hospital (NCH) main campus. Neonates of any GA born between 2012 and 2016 with available urine samples in the Ohio Perinatal Research Network in the first 7 postnatal days were included. Neonates were excluded if they had a documented UTI in the first 7 postnatal days. Patients were classified by GA at birth into 4 categories as follows: < 27, 27–31, 32–35, and ≥ 36 weeks. Informed consent was obtained from parents or legal guardians at the time of sample collection for all the enrolled neonates. The study was approved by the Nationwide Children’s Hospital (NCH) Institutional Review Board (IRB). All experiments were carried out in accordance with the NCH IRB guidelines and regulations.

### Urine samples

Samples were obtained from neonates within the first 7 days of life who had no documented UTI within those first 7 days. The urine samples were collected for research purposes via indwelling urethral catheter when one was present or more commonly via cotton balls placed in the patient’s diaper. Urine was squeezed from cotton balls with a syringe then immediately stored at − 80 °C in the lab until ready for use. Urine samples were then thawed and centrifuged (10 min 350 g 4 °C). Cell–free supernatants were subject to commercial ELISAs to quantify BD-1 (Peprotech, Rocky Hill, NJ) and RNase 7 (Cloud Clone Corp, Katy, TX). Those samples whose ELISA results interpolated with the standard curves for each AMP were included for statistical analysis. AMP levels (ng/ml) were normalized to urine creatinine (UCr, mg/ml) to control for differences in urine concentration. AMP levels are expressed per unit volume (ng/ml) or normalized to UCr (ng/mg). Raw data are provided in the supplemental material.

### Statistical analysis

Descriptive statistics (median, range, frequency, and percent) were summarized for patient demographics and clinical characteristics. Fisher’s exact test was used for testing the association of categorical variables with GA-groups and the Kruskal–Wallis rank sum test was used for detecting differences in distribution of continuous variables by GA-groups. Wilcoxon’s signed rank test was used to test for difference in distribution between log transformed RNase 7/UCr and BD-1/UCr concentrations. Spearman’s correlation was used to summarize the relationship between each AMP with GA or birthweight. Statistical significance was assessed at α = 0.05. All data preparation and analyses were completed using R version 4.3.0.

## Results

### RNase 7 and BD-1 are present in neonatal urine as early as 22 weeks of gestation

A total of 79 newborns with urine samples were identified. Of these newborns, 66 had AMP absorbance values that interpolated on the standard curve for RNase 7 or BD-1 and were therefore deemed suitable for analysis. A total of 31 newborns (47%) had both AMPs detected, 29 (44%) had only RNase 7 detected, and 6 (9%) had only BD-1 detected. The majority of newborns were male (59%) and GA ranged from 22 to 39 weeks. The characteristics and AMP concentrations by GA groups of the included patients are shown in Table 1.

**Table 1:** Patient characteristics, RNase 7, and BD-1 concentrations by gestational age.

Characteristic	< 27 weeks, N = 19^a^	27–31 weeks, N = 17^a^	32–35 weeks, N = 15^a^	≥ 36 weeks, N = 15^a^	*p*-value^b^	Overall, N = 66^a^
Sex					0.062	
Male	8 (42%)	14 (82%)	7 (47%)	10 (67%)		39 (59%)
Female	11 (58%)	3 (18%)	8 (53%)	5 (33%)		27 (41%)
Birthweight (kg)	0.68 (0.57, 0.76)	1.10 (1.00, 1.33)	2.41 (2.11, 2.81)	3.10 (2.85, 3.62)	**< 0.001**	1.37 (0.80, 2.77)
BD-1 (ng/mL)	31 (15, 46)	14 (11, 16)	12 (10, 14)	5 (2, 6)	**< 0.001**	14 (7, 21)
(N Missing)	6	10	7	6		29
BD-1 (ng/mg UCr)	166 (151, 614)	115 (87, 170)	108 (94, 166)	14 (10, 58)	**0.002**	116 (71, 199)
(N Missing)	6	10	7	6		29
RNase 7 (ng/mL)	43 (19, 72)	29 (17, 44)	42 (14, 51)	23 (9, 39)	0.3	32 (14, 58)
(N Missing)	4	1	1	0		6
RNase 7 (ng/mg UCr)	569 (307, 860)	308 (124, 461)	254 (162, 351)	124 (70, 167)	**0.006**	271 (119, 563)
(N Missing)	5	2	2	1		10

^a^n (%); Median (IQR).

^b^Fisher’s exact test; Kruskal–Wallis rank sum test.

Significant values are in bold.

**Figure 1:**
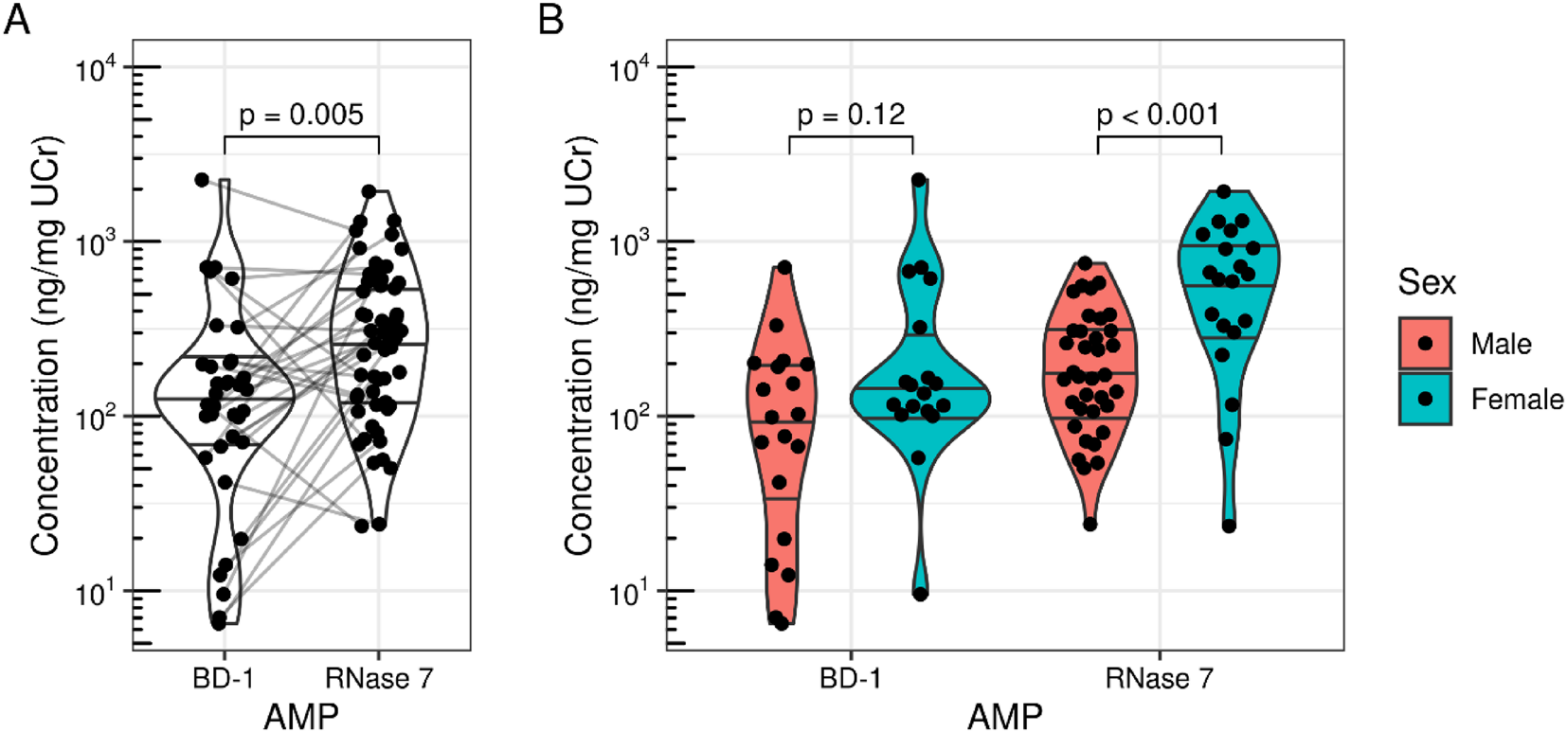
Overall urinary RNase 7 (ng/mg UCr) and BD-1 (ng/mg UCr) (**A**) and concentrations grouped by sex (**B**).

On average, RNase 7/UCr is present in greater quantities than BD-1/UCr (*p* = 0.005). The median urinary concentrations of RNase 7/UCr and BD-1/UCr were 271 ng/mg (IQR: 119, 563) and 116 ng/mg (IQR: 71, 199), respectively (Fig. 1A). Average RNase 7/UCr urinary concentration was significantly higher in females compared to males (*p* < 0.001) (Fig. 1B). There was no statistically significant difference in average BD-1/UCr concentration between female and male neonates (*p* = 0.12). The crude urinary concentrations of RNase 7 and BD-1 across GA and birthweight groups are depicted in Supplemental Fig. 1.

### Urinary RNase 7 and BD-1 concentrations decrease with increasing gestational age

**Figure 2:**
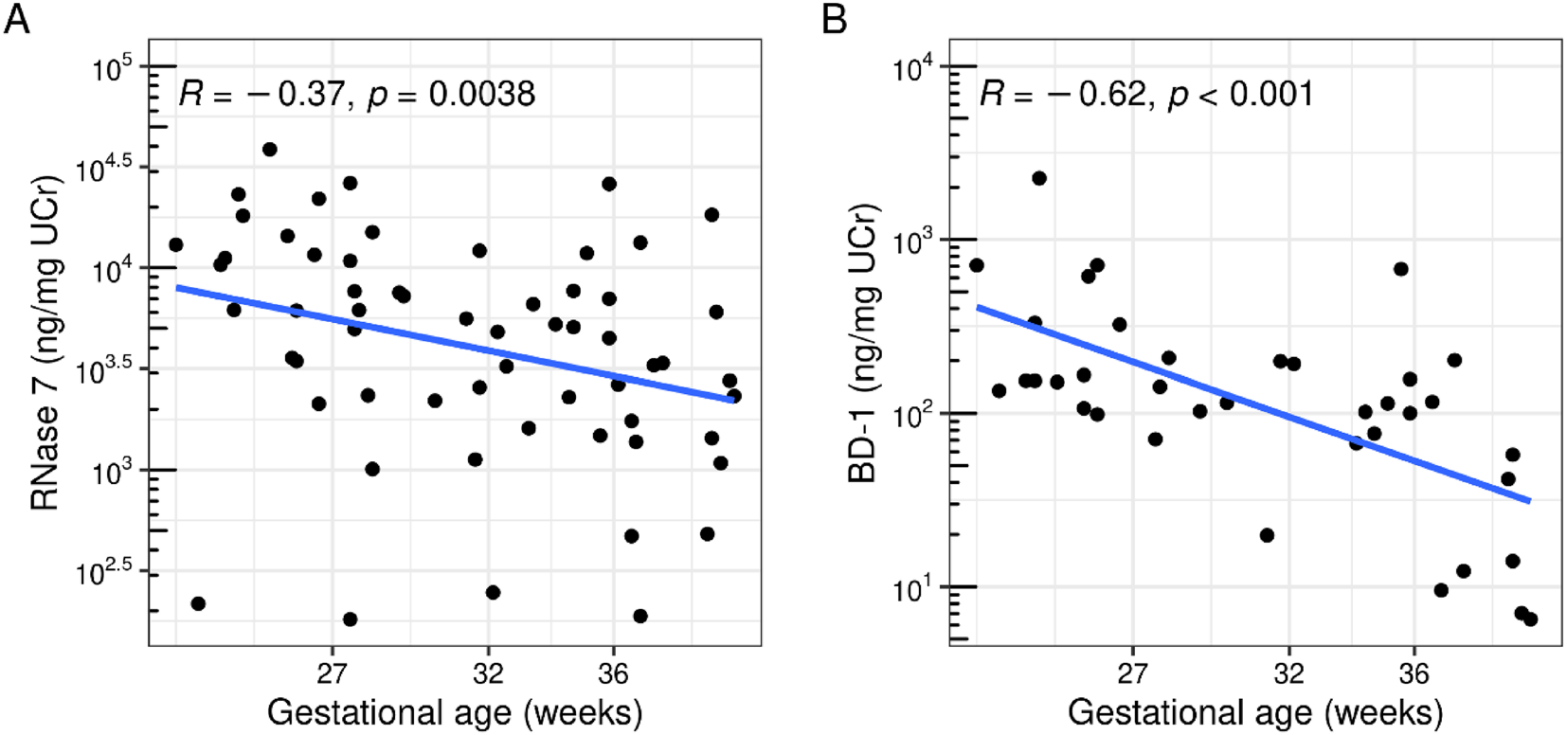
Relationship between gestational age (weeks) and RNase 7 (ng/mg UCr) or BD-1 (ng/mg UCr). Spearman’s correlation (R) and linear regression line for log-scaled AMP values are shown.

**Figure 3:**
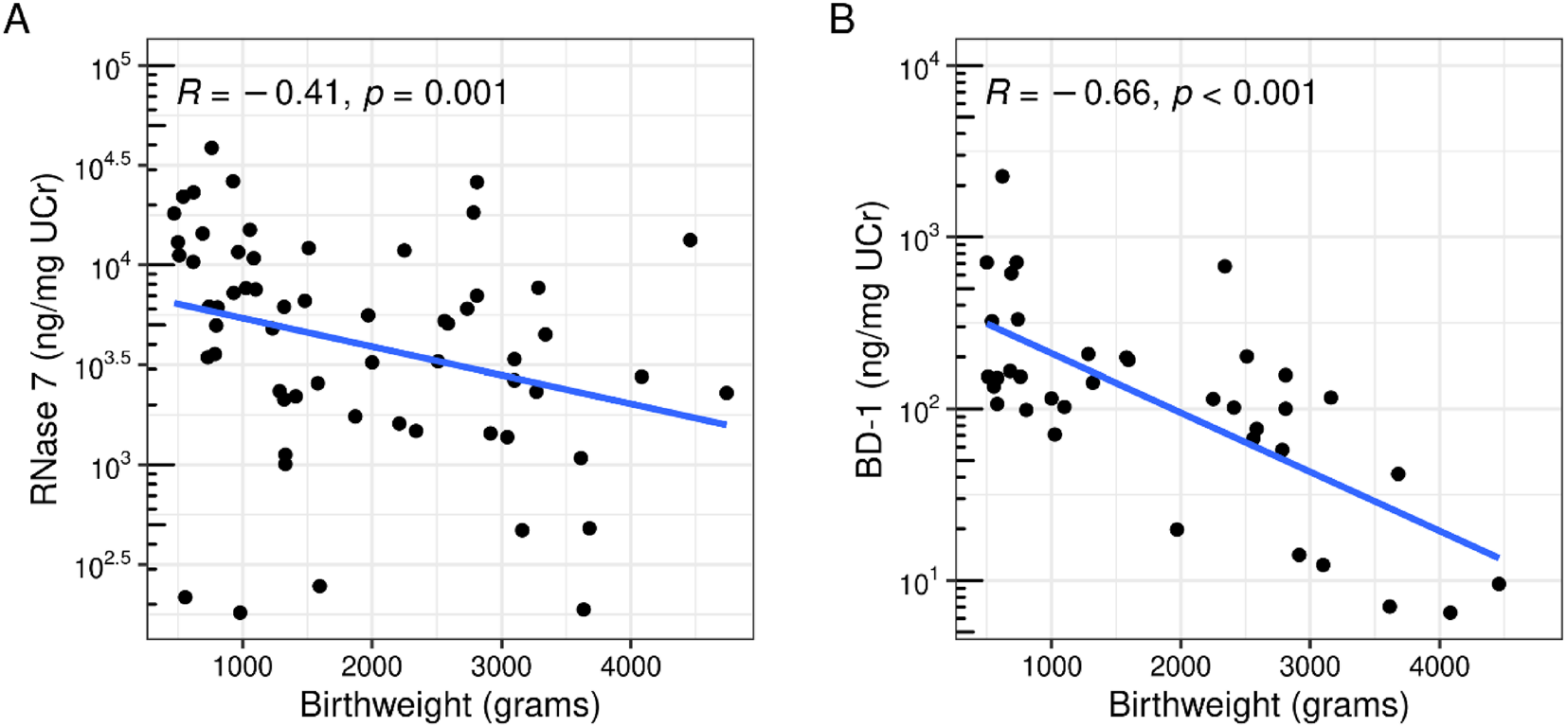
Relationship between birthweight (grams) and RNase 7 (ng/mg UCr) or BD-1 (ng/mg UCr). Spearman’s correlation (R) and linear regression line for log-scaled AMP values are shown.

The concentrations of both RNase 7/UCr (R = − 0.47, *p* < 0.001) and BD-1/UCr (R = − 0.62, *p* < 0.001) decreased with increasing GA (Fig. 2). Because GA and birthweight were highly correlated (R = 0.94, *p* < 0.001) a similar relationship was observed for both AMPs with birthweight (RNase 7/UCr: R = − 0.49, *p* < 0.001 and BD-1/UCr: R = − 0.66, *p* < 0.001) (Fig. 3).

## Discussion

To our knowledge this is the first study that identified the presence of RNase 7 and BD-1 in neonatal urine and characterized the effect of GA and birthweight on AMP concentration. We detected RNase 7 and BD-1 in the neonatal urine even in those neonates who were born at the limits of viability at 22 weeks of gestation. The neonatal urinary concentrations of RNase 7 and BD-1 decreased with increasing GA and birthweight and were higher than the reported levels in older pediatric patients and adults. The urinary concentration of RNase 7 was higher in female compared to male neonates.

The urinary concentration of NGAL, another AMP, has been shown to be higher in neonates compared to older pediatric patients and adults and has an inverse relationship with GA and birthweight^9,10^. Similarly, we found that BD-1 and RNase 7 levels decreased as birthweight increased. A correlation or lack thereof between BD-1 and RNase 7 concentrations has not been investigated previously and the patterns of AMPs concentration in neonates with small, appropriate, or large weight for GA remain to be evaluated.

The diagnosis of UTI in the pediatric and neonatal population has several limitations including lack of specificity for the urinalysis and the invasive nature of obtaining sterile urinary samples for culture. Measuring the concentration of urinary AMPs could be utilized to improve the speed and accuracy of UTI diagnosis in children as recently shown^17^. One of the strengths of this study is the ability to identify the presence and baseline concentration values of neonatal urinary AMPs and characterize the variation by GA and birthweight. This could be the first step in utilizing AMPs as novel biomarkers to improve neonatal UTI diagnostics.

One limitation of our study is the method of neonatal urine collection which involved placement of cotton balls in the diaper. AMPs, as discussed previously, are made throughout the body, including the skin^6,8,11–13^. Therefore, our measured urinary AMPs may possibly be reflective of dermal RNase 7 and BD-1. At this time there is no means of separating out those AMPs made by the skin from those made within the GU tract without obtaining urinary samples directly via catheterization. Since previous literature demonstrated that the GU tract is the major source of both RNase 7 and BD-1 in children and adults^8,11^, we assumed for this study that the AMPs we measured were of GU tract origin rather than dermal origin. Future studies should address whether AMP concentrations could vary based on the urine collection methods. Another limitation is that neonatal eGFR, urine osmolality and urine creatinine are physiologically lower compared to older children^18^. We elected to normalize AMP concentrations to urine creatinine only and speculated that similar patterns will be noticed if AMP concentrations were normalized with eGFR and/or urine osmolality.

This study confirmed the presence of RNase 7 and BD-1 in neonatal urine, defined the overall median concentrations and outlined their variation with GA and birthweight. These findings represent initial necessary steps toward the eventual implementation of novel biomarkers such as AMPs in improving neonatal UTI diagnostics.

### Supplementary Information

Supplementary Figure 1.

## Data Availability

All data generated or analyzed during this study are included in this published article (and its supplementary information files).
