# Supplementary material for: Concentration of novel urinary tract infection biomarkers in neonates
Source: Sci Rep. 2024 Feb 6;14:2996. doi: 10.1038/s41598-024-53486-2 (PMC10844638; doi:10.1038/s41598-024-53486-2)
Supplement: Supplementary file 1 — Supplementary Figure 1. [file 41598_2024_53486_MOESM1_ESM.docx]

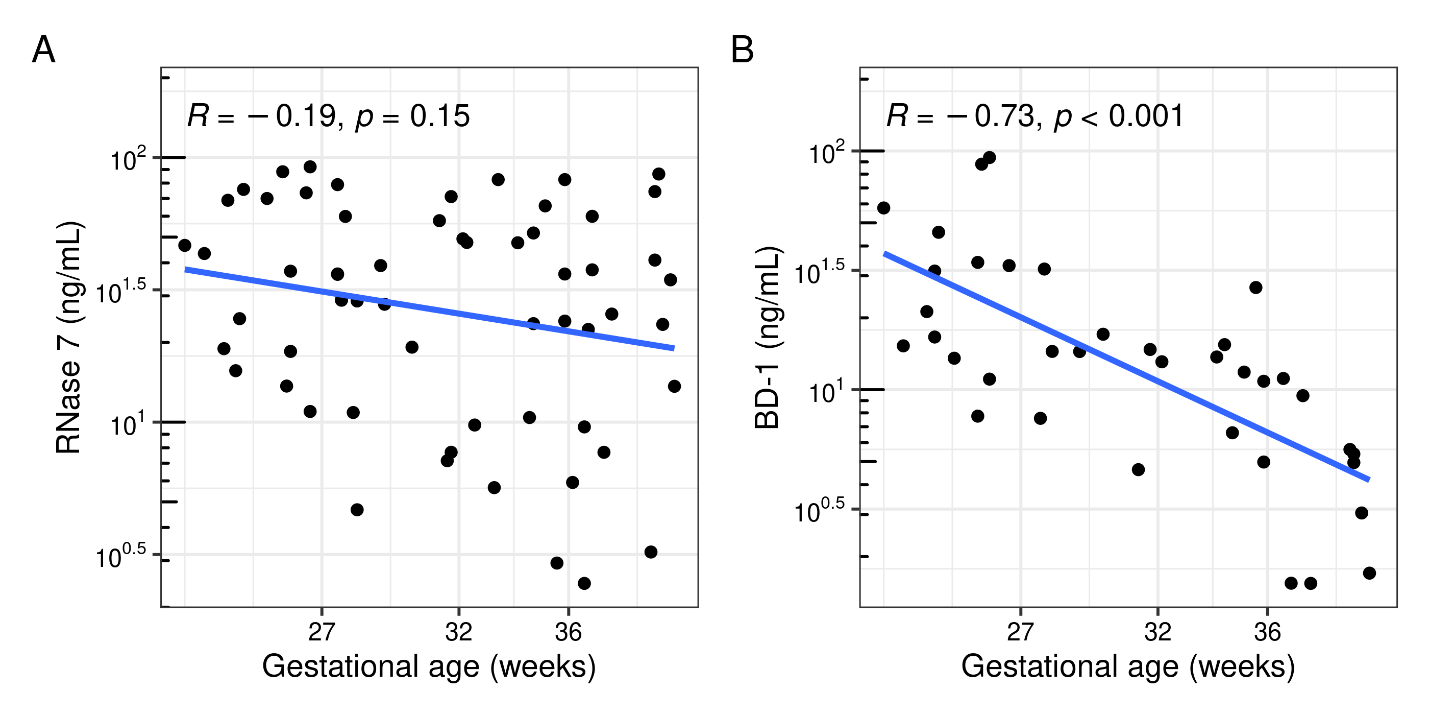

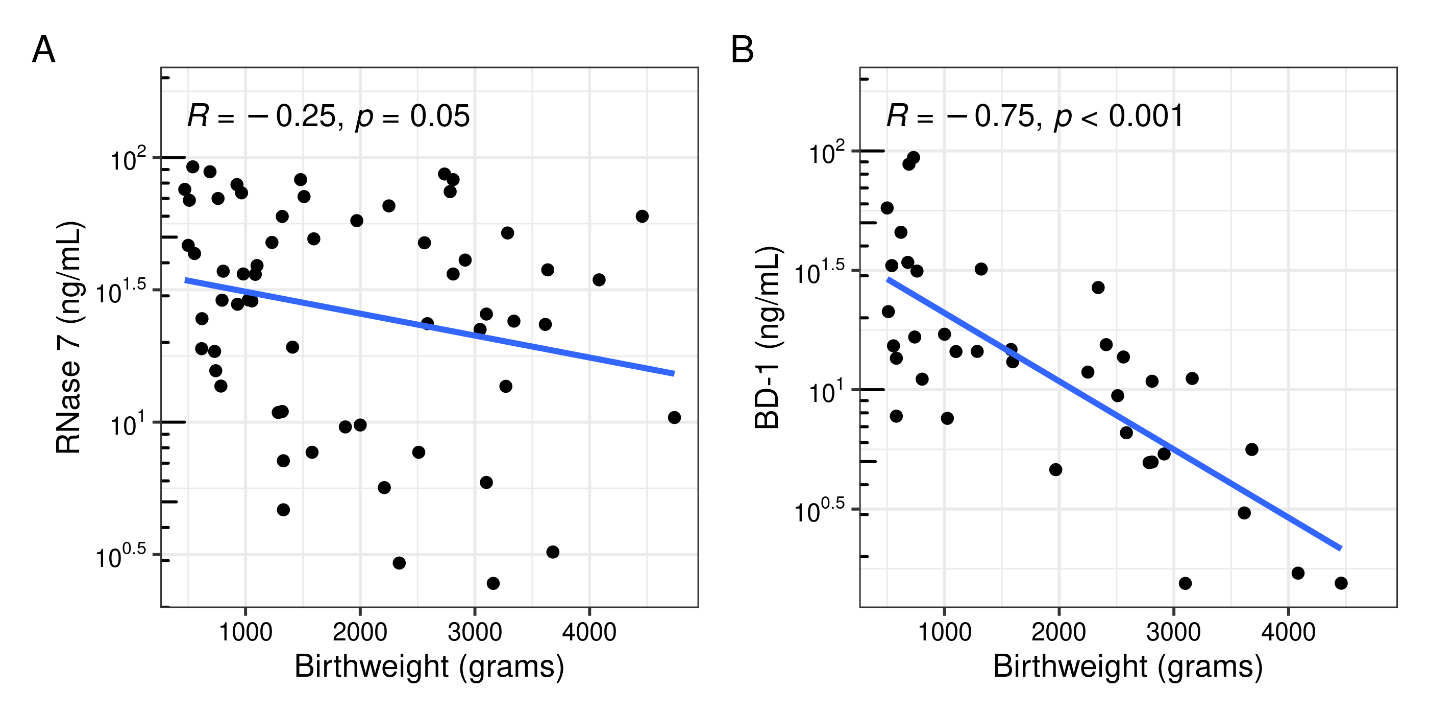


**Supplemental Figure-1**. Relationship between gestational age (weeks) or birthweight (grams) and crude measures of RNase 7 (ng/mL) or BD-1 (ng/mL). Spearman’s correlation (R) and linear regression line for log-scaled AMP values are shown
